# Brain plasticity in aphasic patients: intra- and inter-hemispheric reorganisation of the whole linguistic network probed by N150 and N350 components

**DOI:** 10.1038/srep12541

**Published:** 2015-07-28

**Authors:** Chiara Spironelli, Alessandro Angrilli

**Affiliations:** 1 Department of General Psychology, University of Padova, Via Venezia 8, 35131 Padova, Italy; 2 Center for Cognitive Neuroscience, Padova, Italy; 3 CNR Institute of Neuroscience, Padova, Italy

## Abstract

The present study examined linguistic plastic reorganization of language through Evoked Potentials in a group of 17 non-fluent aphasic patients who had suffered left perisylvian focal lesions, and showed a good linguistic recovery. Language reorganisation was probed with three linguistic tasks (Phonological, Semantic, Orthographic), the early word recognition potential (N150) and the later phonological-related component (N350). Results showed the typical left-lateralised posterior N150 in healthy controls (source: left Fusiform Gyrus), that was bilateral (Semantic) or right sided (Phonological task) in patients (sources: right Inferior/Middle Temporal and Fusiform Gyri). As regards N350, controls revealed different intra- and inter-hemispheric linguistic activation across linguistic tasks, whereas patients exhibited greater activity in left intact sites, anterior and posterior to the damaged area, in all tasks (sources: Superior Frontal Gyri). A comprehensive neurofunctional model is presented, describing how complete intra- and inter-hemispheric reorganisation of the linguistic networks occurs after aphasic damage in the strategically dominant left perisylvian linguistic centres.

The study of human brain plasticity in relation to highly localized functions, such as those underlying somatosensory cortex, has been supported by a consistent animal literature. Instead, language cannot take advantage from animal studies, lacks a “homuncular” organisation, and encompasses almost all cortical structures. Current literature shows that aphasic patients with permanent damage to language areas in the left hemisphere are able to recover their linguistic functions^1^,^2^, potential substitutes including either homologous, right-hemisphere areas^3^,^4^,^5^,^6^, undamaged portions of left-hemisphere linguistic networks^7^,^8^,^9^,^10^ or both^11^,^12^,^13^,^14^,^15^,^16^. Numerous factors, e.g., the site and extent of damaged areas, and patients’ age, education level and motivation, may influence the rehabilitation level achieved and, therefore, affect the functional recovery and the plastic cortical reorganisation of linguistic networks^17^.

Several studies have been carried out to measure both the spatial aspects and the temporal dimension of the reorganisation processes in aphasics, providing evidence that brain plasticity mechanisms lead to the functional linguistic recovery^18^,^19^,^20^,^21^,^22^,^23^,^24^,^25^,^26^,^27^,^28^. As aphasia is an acquired disorder of language due to brain damage, the precise location of lesions can provide essential information not only clarifying brain/behaviour relationships^29^,^30^,^31^,^32^, but also revealing the mechanisms specifically involved in brain plasticity.

In past studies, the earliest components examined in aphasia research were P300/N400 waves^23^,^25^. More recently, ERP/MEG studies of healthy participants have shown that written words systematically evoke electrical/magnetic waves with very short time latencies^33^,^34^,^35^,^36^,^37^,^38^,^39^,^40^,^41^,^42^,^43^, supporting the view that N150, a cortical wave which peaks at about 140–200 ms over left occipito-temporal regions, represents the earliest component—influenced by perceptual learning^33^ – which can reliably distinguish between word-like strings and other visual stimuli. Within neuroimaging studies that showed the distribution of cortical networks involved in various components of written word processing^44^, Dehaene and colleagues^45^ proposed that the Visual Word Form Area—i.e., the left Fusiform Gyrus—is the most crucial area for the structural, pre-lexical representation of a word as an ordered sequence of abstract letters, regardless of size, font or case^41^,^46^ or position in the visual field^34^, and Allison and co-workers^47^,^48^, studying intracranial recordings of epileptic patients, found category-specific responses to letter strings on the surface of the left fusiform gyrus. N150 has also been used as a marker of altered reading in dyslexic children, and the hemispheric asymmetry of this component is functionally modified by linguistic training and significantly correlated with the extent of language improvement^49^.

Past studies on non-fluent aphasics’ slow cortical potentials revealed consistent reorganisation of both phonological and semantic processes in intact anterior orbitofrontal sites^18^,^26^, whereas the activation found in intact left posterior sites was negatively correlated with linguistic performance, and viewed as dysfunctional^26^. Thus, although posterior linguistic networks are usually intact in non-fluent aphasia, there is evidence that damage to left anterior cortices impairs overall within-hemisphere connectivity and the functioning of posterior, de-afferented linguistic areas^50^,^51^. This view is further supported by increased EEG delta amplitude, a neurological index of cortical inhibition, in intact left posterior sites in a group of non-fluent aphasia patients^52^. The present study aimed at examining language reorganisation in 17 recovered aphasia patients by measuring two linguistic components, i.e., the N150, marking word recognition and typically located above the left occipitotemporal regions, and the N350, marking later phonological processing and located in more anterior sites^41^,^53^. Since language originates from the integration of many processes and the underlying neural circuits are coordinated in an ordered hierarchical sequence, we expected that a lesion in a strategic linguistic centre around the left frontal operculum would affect the distribution and role of residual intact linguistic regions, including the left occipito-temporal cortex, important for word recognition and reading. We expected an inhibited left N150 in aphasics as a consequence of disconnection from hierarchically super-ordered left frontal cortices and disinhibition of right homologous regions. The use of ERPs, lesion location mapping and source location analysis^49^ enabled us to both study the temporal dynamics of linguistic processing—otherwise not possible with metabolic methods – and draw up a general model on how left-hemisphere networks are re-organised after damage and how right posterior regions, following disinhibition, increase their activity.

## Results

### Lesion mapping

**Figure 1:**
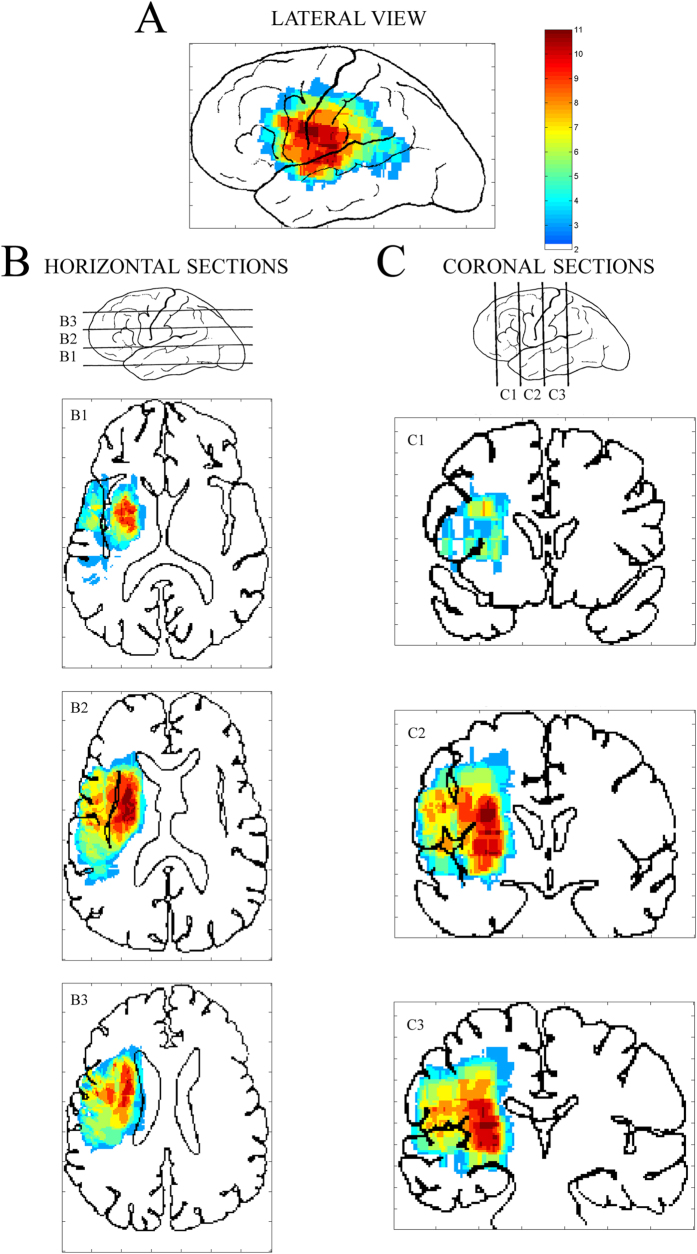
Map of lesions from 17 non-fluent aphasic patients, projected on lateral view of left hemisphere (**A**): colours from pale blue to red mark increasing number of patients with cortical/subcortical lesions. (**B**) Horizontal slides of lesions, projected from lower (B1) to higher (B3) cortical sections. (**C**) Coronal view of lesions, projected from more anterior (C1) to more posterior (C3) sections. Left hemisphere on left of sections.

Figure 1 shows the numbers of overlapping lesions of aphasic patients projected on the left lateral view (Fig. 1A) and on both horizontal (Fig. 1B) and coronal sections (Fig. 1C). The colours, ranging from pale blue to red, mark the increasing number of patients with cortical/subcortical lesions in that voxel.

For most patients, the left lateral view (Fig. 1A) revealed a core injury which comprised the frontal operculum (BA 44), inferior agranular frontal gyrus (BA 6), inferior lateral portion of the pre-central and post-central gyri (BAs 4, 1) and the central portion of the superior temporal gyrus (BAs 41–42). In a few patients, lesions also extended to the pars triangularis (BA 45), posterior granular frontal gyrus (BA 9), anterior supramarginal gyrus (BA 40) and posterior portion of the upper temporal gyrus (BAs 22–42). At subcortical level, horizontal sections (Fig. 1B) showed deep lesions, in most patients affecting the left putamen, insula and frontal operculum (sections B1 and B2), anterior limb of the internal capsule (section B2), head of the caudate nucleus and lower posterior frontal gyrus (sections B2 and B3) and part of the corona radiata (section B3). Coronal views (Fig. 1C) also showed that both the upper longitudinal fasciculus and the lower frontal gyrus were damaged (section C1). In coronal sections, deep injuries to the insula, putamen, internal and external capsules and, partially, the lateral portion of the globus pallidus (section C2) were also evident, as well as critical damage to portions of the corona radiata (sections C2 and C3). Lastly, cortical lesions were more limited than deep white and grey matter and involved the pre- and post-central gyri, superior temporal gyrus and a small anterior portion of the supramarginal gyrus (section C3).

### Behavioural data

Performance analysis revealed slower responses in aphasic patients (mean: 1547 ms) than in controls (mean: 979 ms; Group main effect: *F*(1,32) = 19.86, *P* < 0.001). Overall, RTs were longer in both the Semantic (1493 ms) and Phonological tasks (1364 ms) but not in the Orthographic task (931 ms; Task main effect: *F*(2,64) = 45.09, GG ε = 0.90, *P* < 0.001). The interaction Group by Task (*F*(2,64) = 16.94, GG ε = 0.90, *P* < 0.001) showed that aphasic patients were slower in the Semantic (1904 ms) and Phonological (1734 ms) than in the Orthographic task (1002 ms), whereas controls showed no significant differences among tasks (1082, 993 and 860 ms, respectively). Groups did not reveal any differences in error rates.

### ERP data: Recognition Potential (N150)

**Figure 2:**
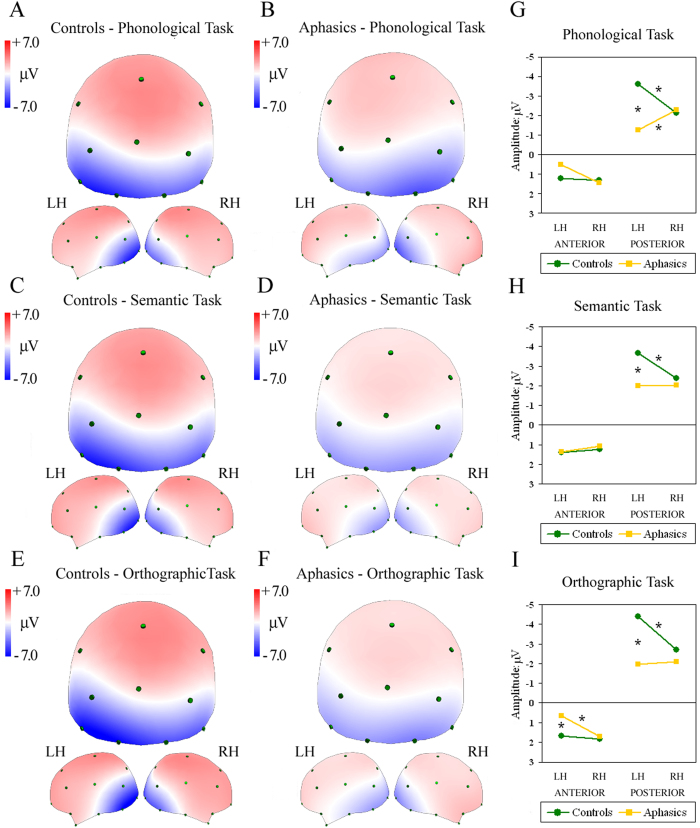
Maps re-referenced to average reference, representing mean potential recorded in 130–150-ms interval during W1 presentation (N150 or Recognition potential). Spline maps of controls (**A**,**C**,**E**) and aphasic patients (**B**,**D**,**F**) during Phonological (**A**,**B**), Semantic (**C**,**D**) and Orthographic tasks (**E**,**F**). Each map shows posterior, left and right views of head. Right: significant four-way Group by Task by AP asymmetry by Laterality interaction, split into (**G**) Phonological, (**H**) Semantic and (**I**) Orthographic data. Asterisks: significant post-hoc tests.

Figure 2 shows ERP spline interpolated maps of controls and aphasic patients during the N150 time interval. In controls, the first component analysed (130–150 ms interval), corresponding to automatic word recognition, was characterised by a negativity peak over left posterior parieto-occipital locations in all tasks (see Fig. 2A,C,E). In contrast, aphasic patients showed different patterns, with bilateral activation over posterior parieto-occipital sites during both Semantic and Orthographic tasks, and a reversed pattern with a negativity peak over right posterior sites for the Phonological task (see Fig. 2B,D,F). This pattern was confirmed by the significant four-way interaction Group by Task by AP asymmetry by Laterality (*F*(2,66) = 3.36, GG ε = 0.91, *P* < 0.05). Post-hoc analysis in the Phonological task revealed greater negativity over left compared with right posterior locations in controls (*P* < 0.001, see green line in Fig. 2G), but greater negativity over right compared with left posterior sites in aphasics (*P* < 0.01, see yellow line in Fig. 2G).

In the Semantic and Orthographic tasks, groups differed significantly in posterior sites (cfr. Fig. 2H,I): controls showed greater negativity over left than right posterior clusters (*P* < 0.001; green line), whereas patients showed bilateral activity (Fig. 2H,I, yellow line). Aphasics also showed greater left vs. right negativity over anterior sites in the Orthographic task (*P* < 0.05, red line in Fig. 2I). In addition to differences in lateralisation patterns, direct comparisons between groups showed significant greater negativity in left posterior clusters in controls compared with aphasic patients in all tasks (*P* < 0.001).

**Figure 3:**
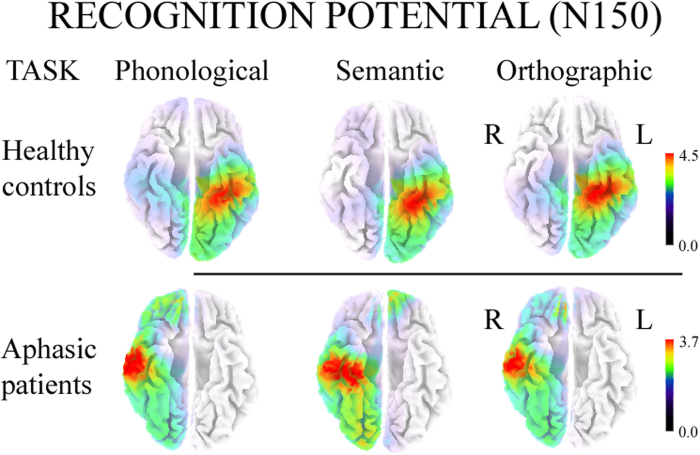
sLORETA source analyses on N150 components evoked by Phonological, Semantic and Orthographic tasks in controls (top) and aphasic patients (bottom). L = Left; R = Right.

sLORETA analyses carried out on all participants revealed significant activity in the N150 interval (130–150 ms after word onset), compared with an interval with no active linguistic processing (20–0 ms before word onset), regardless of task (all *ps* < 0.01). Source analysis of the control group located the main cortical generator of the N150 component (Fig. 3) in the left Fusiform Gyrus (approximate coordinates: Orthographic task −25, −40, 15; Phonological task −30, −40, −19; Semantic task −30, −40, −15). In aphasic patients, the main cortical generator of N150 was found in the right Inferior/Middle Temporal Gyrus (approximate coordinates in Orthographic and Phonological tasks: 57, −18, −22 and 61, −13, −17, respectively) and right Fusiform Gyrus (approximate coordinates in Semantic task: 28, −27, −22).

### ERP data: Phonological processing (N350

**Figure 4:**
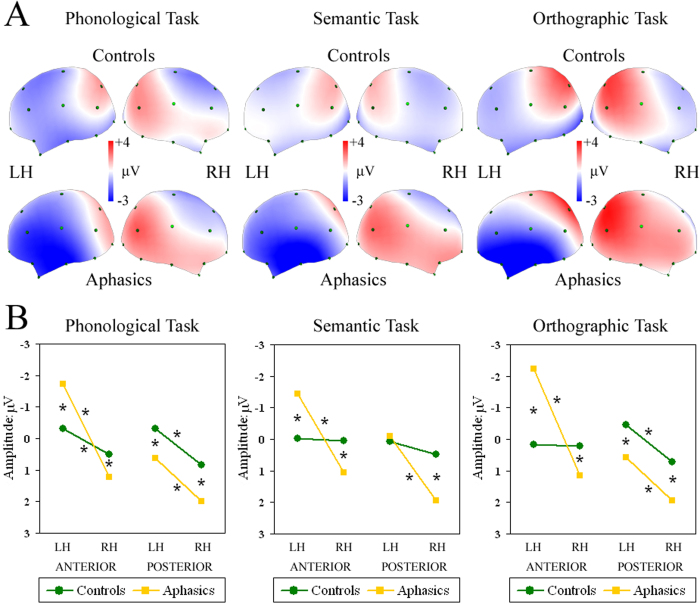
Top (**A**): Maps re-referenced to average reference, representing mean potential recorded in 300–500-ms interval during W1 presentation (N350 or Phonological processing). Spline maps of controls and aphasic patients during Phonological, Semantic and Orthographic tasks. Each map shows left and right views of head. Bottom (**B**): Significant four-way Group by Task by AP asymmetry by Laterality interaction during Phonological, Semantic and Orthographic tasks. Asterisks: significant post-hoc tests.

The N350 interval (300–500 ms) was characterised by differing task- and group-dependent patterns. Controls showed task-specific activation, but aphasics had the same strongly left-lateralised pattern in both anterior and posterior clusters, regardless of task (Fig. 4A). The significant four-way Group by Task by AP asymmetry by Laterality interaction (*F*(2,66) = 7.18, GG ε = 0.85, *P* < 0.01) revealed that, during the Phonological task, controls had significantly greater left vs. right negativity in both anterior (*P* < 0.01) and posterior clusters (*P* < 0.001), during the Semantic task they showed an overall bilateral pattern, and during the Orthographic task they were left-lateralised only in posterior sites (*P* < 0.001; green line in Fig. 4B). Aphasics in all tasks had a greater left vs. right negativity in anterior sites (all *P* < 0.001; yellow line in Fig. 4B), which was significantly larger than that of controls. As regards posterior locations, patients exhibited a significantly greater negativity in left vs. right sites in all tasks (all *P* < 0.001; yellow line in Fig. 4B). In addition, greater positivity was observed in all posterior clusters of patients compared with those of controls, with the exception of the left cluster during semantic processing (all *P* < 0.001; yellow vs. green line in Fig. 4B).

**Figure 5:**
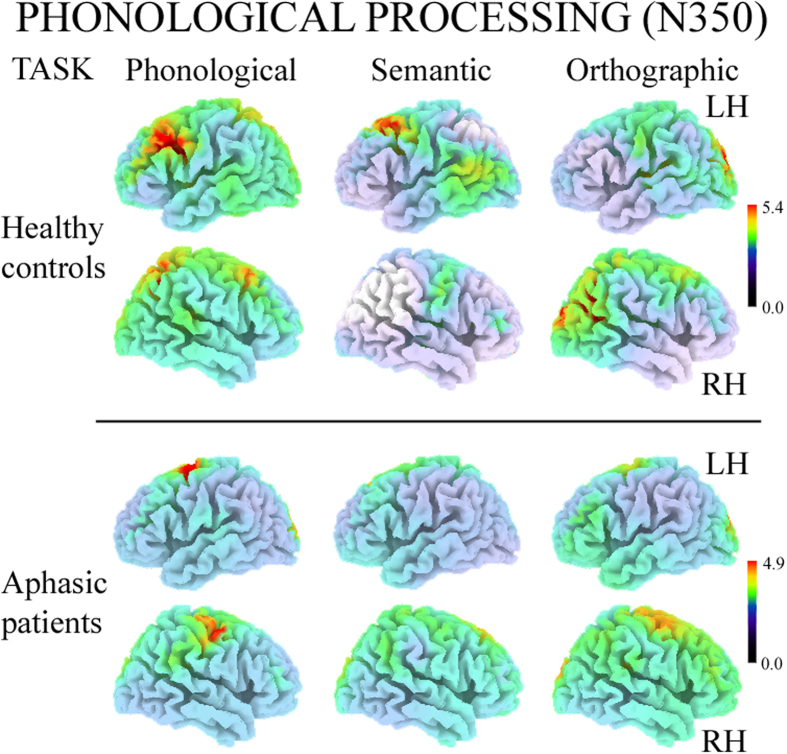
Left and Right hemisphere views of sLORETA source analyses on N350 components evoked by Phonological, Semantic and Orthographic tasks in controls (top two rows) and aphasic patients (bottom rows). LH = Left Hemisphere; RH = Right Hemisphere.

sLORETA analysis carried out on both groups revealed significant activity in the N350 interval (300–500 ms after word onset) compared with an interval with no active linguistic processing (200–0 ms before word onset), regardless of task (all *P* < 0.05). Source analysis of controls located the cortical generator of N350 elicited by the Phonological and Semantic tasks in the left Medial Frontal Gyrus (approximate coordinates: −34, 15, 33; −44, 12, 50, respectively) and in the Orthographic task in the right Superior Temporal Gyrus (approximate coordinates: 36, −57, 29; Fig. 5).

In aphasic patients, the cortical generator of N350 was located in various portions of the left (approximate coordinates of Phonological and Orthographic tasks:: −25, 0, 63; −15, −12, 50) and right Superior Frontal Gyrus (approximate coordinates: Semantic task: 5, 33, 54; Fig. 5).

## Discussion

The present study examined the temporal dynamics of the extended neural network involved in language and alterations in this circuit after anterior left hemispheric damage in non-fluent aphasic patients. We therefore used a well-validated experimental paradigm based on ERPs^18^,^26^,^54^,^55^,^56^,^57^,^58^,^59^,^60^,^61^. In the present study, non-fluent aphasic patients showed a decreased N150 component over the left parieto-occipital cortex with respect to controls, and regular N150 activation over right homologous locations. In particular, only during the Phonological task did patients exhibit significant right lateralisation on posterior electrodes, whereas bilateral activation was found in the Semantic and Orthographic tasks. In healthy controls, the cortical structure generating N150 was mainly located around the left Fusiform Gyrus, matching neuroimaging studies^34^,^45^,^46^, whereas aphasic patients revealed the involvement of similar locations but in the right hemisphere, i.e., the right middle/inferior temporal regions. These findings are important when we recall that all sites included in our statistical analyses were located over intact (unimpaired) cortical areas. The disruption of intra-hemispheric connections between anterior damaged areas and posterior undamaged portions of the residual linguistic networks causes reorganisation of all word processing, also involving linguistic processes far from the damaged areas. According to Hagoort^62^ and Bookheimer^63^ the left Inferior Frontal Gyrus (IFG)—including BAs 44–45 (i.e., Broca’s area), BA 47 and the ventral portion of BA 6—plays a crucial role in the integration of all processes throughout the linguistic network. The core role played by the left IFG in unification becomes clear, for example, when we see that it can organise all relevant semantic-lexical information into a consistent response. Semantic categories are linked to their critical features^64^,^65^ and distributed at the level of the temporal lobes in both hemispheres^54^,^57^,^66^,^67^,^68^,^69^. Thus, significant damage to the area around the left IFG may dramatically alter the functioning of the whole linguistic network, even for processes in cortical regions far from the lesion.

The plastic reorganisation of language was functionally different in the later phase of word reading, characterised by N350 over left frontal sites^41^,^53^. In this time interval, controls exhibited greater left negativity in both anterior and posterior sites during the Phonological, bilateral activation during the Semantic, and larger left posterior negativity during the Orthographic task. Source analysis of N350 in controls showed the involvement of various portions of the left Medial Frontal Gyri during phonological and semantic processing, and right temporo-parietal regions during the visuo-perceptual control task, matching Hagoort’s unification model, in which the regions around the left lateral prefrontal cortex play a dominant role in the integration of all linguistic (but not visuo-perceptual) information^62^. Aphasic patients, unlike controls, exhibited the same pattern of activation in all tasks, showing not only greater negativity on left intact sites located anterior to the damaged area, but also relatively greater left lateralisation over posterior sites. Source analysis of N350 revealed the involvement of various portions of the left Superior Frontal Gyri, regardless of task. This finding suggests that controls activate different linguistic networks for each specific linguistic task, whereas aphasic patients mainly use left anterior areas around the lesion, over-activating these regions not only for phonological but for all linguistic processes, indistinctly, and exhibiting generalisation of recovered linguistic processes. This result is in line with past studies carried out with slow evoked potentials^18^,^26^ which revealed that the new network operates independently of the specific process required, i.e., the same left-lateralised orbitofrontal areas were involved during both Phonological and Semantic tasks, showing generalisation of linguistic processing and over-use of spared linguistic areas for the main linguistic functions.

**Figure 6:**
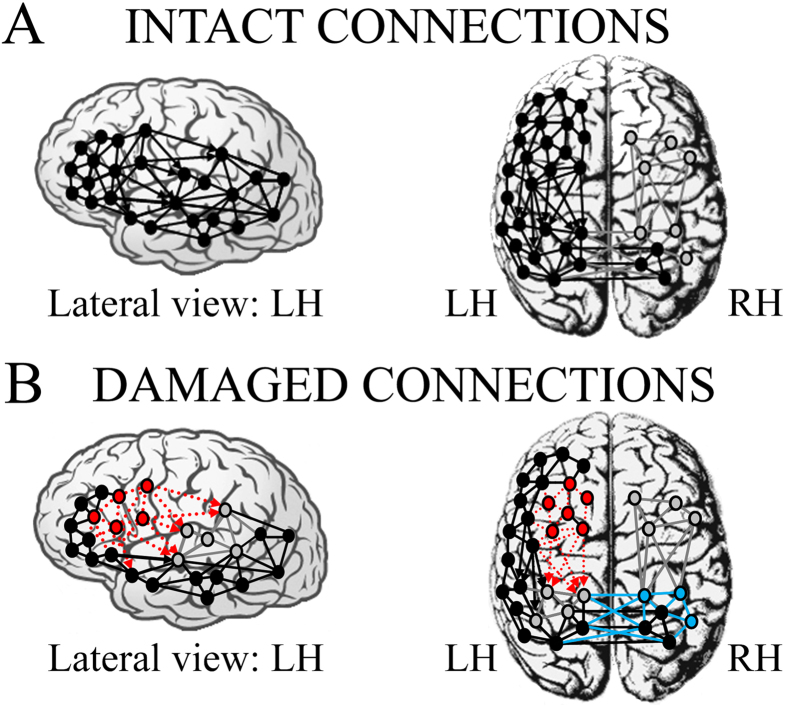
Intra- and inter-hemispheric connections presumed/supposed to represent neural network model activated by linguistic circuits in (**A**) healthy controls and (**B**) non-fluent aphasic patients. Black nodes and connections: intact neural components; grey nodes and connections: (**A**) silent or less active centres in right hemisphere due to left-dominance for language and (**B**) inhibited/deafferented or less active centres in posterior left hemisphere due to the lesion on the main anterior linguistic centre. Red circles and dotted arrows (**B**): damaged non-functional areas. Cyan circles and arrows (**B**): re-activation or more active centres in right hemisphere as consequence of the lack of contralateral inhibition of posterior left centres (in grey) after deafferentation from damaged non-functional anterior areas (in red).

On the basis of present and past research on language and aphasic patients, a model of the intra- and inter-hemispheric connections presumed to represent the linguistic network re-organised after focal perisylvian lesions can now be drawn up (Fig. 6). Two main linguistic centres can be identified in a normal unimpaired brain: an anterior dominant network, related to the organisation of all linguistic processing and the components of speech production, and a more posterior network, related to the comprehension constituents of language (Fig. 6A, lateral view). According to Wernicke-Geschwind model^70^ each network arises from the close interconnection of several critical areas in the left hemisphere, such as Broca’s area (*pars triangularis* and *pars orbitalis*), the inferior frontal gyrus and insula for the anterior centre, and Wernicke’s area, inferior and middle temporal gyri, and fusiform and lingual gyri for the posterior centre. Both centres are also highly interconnected within the same hemisphere, both by direct subcortical connections (see arrows in Fig. 6A) and by indirect short cortical connections.

The posterior network of the left hemisphere is directly connected to a parallel linguistic network in the right hemisphere (Fig. 6A, top) and, together, they represent an extensive bilateral circuit for comprehension and lexical-semantic representation (e.g., speech comprehension or reading). With respect to the anterior left network, which plays a dominant role in integrating all linguistic information^62^, the homologous cluster of the right hemisphere has limited linguistic capacity and is relatively less functionally connected (for language) to the posterior network (Fig. 6A, right panel, in grey). A lesion centred over strategic left anterior perisylvian regions can disrupt this complex network (Fig. 6B, in red) and force functional reorganisation of all word reading dynamics^18^,^26^. Thus, the lesion not only produces a functional disconnection between the residual cortical areas belonging to the same dominant linguistic hemisphere (Fig. 6B, in red), but also the loss of dominant frontal afferents, leading to inhibition of intact, more posterior regions (dashed red arrows in Fig. 6B). Therefore, a significant alteration also occurs in linguistic processes which generally involve intact cortical structures functionally connected and down-regulated by the anterior damaged ones. This relative left posterior inhibition reduces control over right posterior cortices, which in turn tend to compensate and over-activate during early phases of word recognition (Fig. 6B, in cyan). The undamaged, but disconnected, left posterior regions of our aphasics failed to show the typical left dominant N150 observed in controls, and this disruption led to disinhibition of contralateral right sites in which the main N150 generator was found.

The results of N350 analysis match well the above described model. We hypothesise that, after substantial damage to a portion of the dominant left frontal linguistic centre, which typically shows functional differentiation across various processes (see N350 in controls)^63^, the residual intact areas anterior to the lesion are still hierarchically dominant but over a smaller re-structured network, which is no longer able to organise linguistic processes in efficient and specialised sub-networks. This leads to generalisation of the activation and loss of specificity of spared prefrontal sites anterior to the core lesion, an after-effect of the preserved dominant role of left anterior centre, but the limited extension of this area probably leads to reduction of the original extended linguistic networks. The anterior and posterior linguistic centres can then communicate by means of undamaged short cortical intra-hemispheric connections. However, this second pathway is slower and less effective than that of the damaged subcortical long-tract fibres.

This hierarchical linguistic model is further supported by converging evidence from several other studies in which the N150 probe was sensitive to a disrupted left hemisphere network in patients with a variety of impairments: cognitive (i.e., dyslexic children^49^), psychiatric (i.e., schizophrenia patients^61^,^71^,^72^) and neurodegenerative disorders (i.e., Alzheimer disease patients^73^).

With respect to neurological lesions affecting well-organised and spatially confined cortical functions (e.g., somatosensory, motor or visual^74^,^75^,^76^), damage to the extended linguistic neural circuit has in principle a larger recovery potential and greater plasticity in the reorganisation of lost features. Nevertheless, because of the strong hierarchy among linguistic regions, not all lesions of this extended network are equivalent, and a lesion in the dominant left frontal centre tends to alter the whole linguistic circuit, including distant intact sites. The approach used in the present study allowed to investigate statistical patterns of recovery common to a sample of patients with similar lesions. Using a complementary approach, it would be interesting also to investigate the variety of language recovery trajectories in patients with similar lesions^77^. Knowledge of the complex interactions and functional interconnections between and within the linguistic centres will allow better targeting of recovery strategies in aphasia. In the present study, N150 represented a valid probe to assess the integrity and reorganisation of the linguistic cerebral architecture in its entirety.

## Methods

### Participants

Seventeen aphasic patients (nine women and eight men, mean age 49.35 ± 14.8 years, mean education 10.8 years) were recruited from A.IT.A (*Associazione ITaliana Afasici,* Italian Aphasics Association), Padova section. All patients had suffered a single cerebrovascular accident of the left hemisphere involving the perisylvian cortex, the most important region for linguistic processing. The average time since the vascular event was 44.5 months (range: 6–198 months; see Table 1 for details).

**Figure 7:**
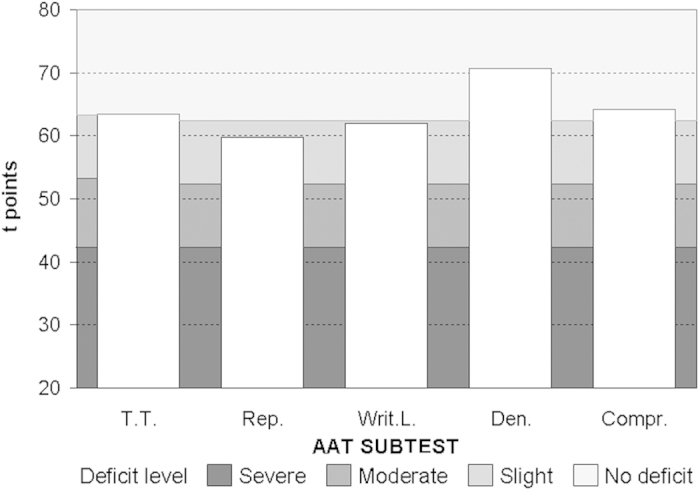
Averaged T (transformed) scores of aphasic patients in each AAT subtest. T.T. = Token Test; Rep. = Repetition; Writ.L. = Written Language; Den. = Denomination; Compr. = Comprehension.

Patients were diagnosed as non-fluent aphasics during the acute phase, on the basis of both CT/MRI documentation of the cortical lesion and their neurological symptoms. Prior to the experimental session, all patients were also tested for language deficits with the Aachen Aphasie Test (AAT) validated for the Italian language^78^,^79^. In AAT subtests, aphasic patients demonstrated average intact language or very mild deficit at the time of the experiment, thus revealing substantial recovery of linguistic functions (see Table 1 and Fig. 7).

Patients’ lesion maps were drawn according to the method of Damasio & Damasio^80^, starting from individual cortical CT or MRI scans of each patient. The scans were then carefully mapped onto standard templates ranging from A1 to A4 scan slopes according to the criteria recommended by Damasio & Damasio^80^. Individual patient templates were then combined by an *ad hoc* Matlab program, implemented for averaging individual templates in inclusive lesion mapping. In the final step, the program drew a map in which each voxel represented a colour corresponding to lesion density (i.e., the number of lesions falling within that specific voxel, a measure which ranged from 2 to 11 lesions in the final, coloured map; see Fig. 1).

Eighteen healthy volunteers, matched for gender (ten men, eight women), age (mean: 55.55 ± 10.2 years) and education level (mean: 11.5 years) served as the control group. Patients were on average 93% right-handed, according to the Edinburgh Handedness Inventory^81^ before the cerebral accident; healthy controls were on average 96% right-handed. All participants also performed a digit span test to verify their short-term memory capacity: this was necessary because our paradigm (W1-W2) required a relatively good level of (verbal) working memory. Patients showed an average digit span of 4.56 and controls 6.00 (*t*(33) = 4.20, *p* < 0.001), i.e., the deficit in patients’ working memory was present, but not so severe to compromise their task execution.

All participants gave their written informed consent to the study, according to the Declaration of Helsinki. Experimental procedures were approved by the Ethics Committee of the Department of General Psychology (University of Padova, Italy).

### Materials

EEG cortical activity was recorded from 26 tin electrodes, 19 placed on an elastic cap (Electrocap) according to the International 10–20 system^82^ and the other 7 applied below each eye (Io1, Io2), on the two external canthi (F9, F10), nasion (Nz) and mastoids (M1, M2). All cortical sites were on-line referred to Cz. Data were stored with Acquire NeuroScan software, version 4.1. Amplitude resolution was 0.1 μV; bandwidth ranged from DC to 100 Hz (6 dB/octave). Sampling rate was set at 250 Hz and impedance was kept below 5 KΩ. After modelling eye movements artifacts (see below) through individual eye calibration, all external electrodes become active free-of-artifacts EEG sites, therefore for source localization carried out with sLORETA, 26 electrode sites including 7 electrodes in the orbitofrontal sites have been used. Although this seems a small number of electrodes to provide a reliable source localization, sLORETA is effective with respect to other methods in locating sources also with standard 32-site montage, although with reduced precision compared with high density recordings^83^,^84^. Furthermore, the present validated paradigm was optimized starting from past studies which localized the N150 source in the fusyform gyrus both with intracranial recording on 34 epileptic patients^47^,^48^, and with a combination of high density EEG with MRI and fMRI in healthy subjects^34^,^35^.

Stimuli consisted of bi- or trisyllabic Italian content words selected from a frequency dictionary of 5000 written Italian words^85^. Words were presented in pairs on a 17” PC screen one at time, with an interstimulus interval of 2 seconds (s): the first word (W1) remained on the screen for 1 s, the second word (W2, or target word) until the participant responded by pressing a keyboard button, but in any case no longer than 5 s. Word pairs were administered in three separate blocks, which corresponded to three linguistic tasks: thus, the same words were presented as W1, but in a different randomised order. In the Phonological task, upon W2 target presentation, participants were asked to decide whether the word pairs rhymed (e.g., brodo-chiodo [broth-nail]) or not (e.g., sabbia-poltrona [sand-armchair]). In the Semantic task, they were asked to judge whether target word W2 was semantically related to the previous word (e.g., brodo-minestra [broth-soup]) or not (e.g., sabbia-denaro [sand-money]). In the Orthographic task, which served as a control task, participants were asked to decide whether the word pairs were written in the same case (e.g. the pair BRODO-FRUTTA or stella-braccio [BROTH-FRUIT or star-arm]) or not (e.g., sabbia-LIBRO [sand-BOOK]). We considered this latter a control task since the same list of words of both Phonological and Semantic tasks was used, but a simple visuo-perceptual matching was requested. Thus, the automatic word processing (N150) should be the same evoked during rhyming and semantic processing, but the later word analysis (corresponding to the N350 component) is expected to reflect the required task-related visuo-perceptual processing.

Participants pressed the keyboard button with the index or middle finger of their left hand to indicate their responses. Each task included 80 trials/word-pairs. In all tasks, 50% matches were randomly interspersed with 50% mismatch trials. The order of the tasks was randomly varied across subjects.

### Data analysis

Error rates and mean Response Times (RTs) of each participant served as behavioural measures, and mean performance was compared between groups and among tasks. EEG was continuously recorded in DC mode, and stored for analysis. Data were off-line re-referenced to the average reference, and subdivided into 1200-ms intervals, including 200 ms before and 1000 ms after W1. A 100-ms baseline preceding W1 was subtracted from the whole trial epoch. Single trials were corrected for eye movement artifacts, i.e., vertical and horizontal movements and blinking, with BESA software (Brain Electrical Source Analysis, version 5.1) in order to compute ocular correction coefficients according to Berg and Scherg^86^,^87^. This correction, based on eye movement modelling, can retrieve clean EEG activity from all electrodes around the eyes. Each trial was then visually inspected for residual artifacts and, if necessary, rejected. After this step, all accepted trials were averaged for each task and participant.

Starting from the time-intervals used in past research^41^,^53^ and from grand-average waveforms, two epochs entered statistical comparisons: one was within a time window around the N150 wave, i.e., in the 130–150-ms interval after W1 onset. The second epoch included the mean potential measured during the N350 wave (interval 300–500 ms). Electrodes were clustered into four regions of interest to carry out statistics with two spatial factors of two levels each: antero-posterior asymmetry and laterality. Every quadrant included three electrodes: Anterior Left (AL: Fp1, F9, F7), Anterior Right (AR: Fp2, F10, F8), Posterior Left (PL: P3, P7, O1) and Posterior Right (PR: P4, P8, O2). Orbitofrontal electrodes (Fp1, Fp2, F9, F10, Nz, Io1, Io2) are generally used to detect or correct eye movements but, after the eye correction method of Berg and Scherg^86^,^87^ had been applied, these electrodes can be considered active, artifact-free cortical sites.

For statistical purposes, behavioural and electrophysiological data have been preliminarily analysed to be sure that data had a normal distribution (by means of both Kolmogorov-Smirnov *d*s and Q-Q plots): for this reason, all statistics were carried out using parametric test, i.e., the Analysis of Variance (ANOVA).

As regards behavioural measures (mean error rates and RTs), ANOVAs were carried out by contrasting the between-subjects Group factor (two levels: Controls vs. Aphasics) and the within-subjects Task factor (three levels: Orthographic vs. Phonological vs. Semantic task). For electrophysiological data (N150 and N350 components), ANOVAs included the following variables in each time window: Group (two levels: Controls vs. Aphasics), Task (three levels: Orthographic vs. Phonological vs. Semantic), AP asymmetry (Anterior-Posterior asymmetry, two levels: Anterior vs. Posterior), and Laterality (two levels: Left vs. Right side). Post-hoc comparisons were computed by the Newman-Keuls test, and Greenhouse-Geisser correction was applied when necessary (df > 2).

In order to identify neural sources underlying word reading, the distributed source solution of every task-related N150 and N350 component by sLORETA (standardised Low Resolution Electromagnetic Tomography)^88^ was compared separately for the two groups. Since sLORETA computes the smoothest possible 3D-distributed current source density solution constrained to grey matter, this approach was particularly suited for our analysis since, due to the smoothness constraint, it does not need an *a priori* definition of known sources. As in a prior study^49^, separate *t*-tests were carried out for each task and group by comparing electrical activity within the N150 interval (130–150 ms after word onset) with that of an interval with no active linguistic processing (the 20-ms baseline prior to word onset). The N350 interval (300–500 ms) was also compared with an equivalent neutral interval (i.e., the 200-ms baseline). All results are expressed in Talairach coordinates^89^.

## Additional Information

**How to cite this article**: Spironelli, C. and Angrilli, A. Brain plasticity in aphasic patients: intra- and inter-hemispheric reorganisation of the whole linguistic network probed by N150 and N350 components. *Sci. Rep.* **5**, 12541; doi: 10.1038/srep12541 (2015).

## Figures and Tables

**Table 1:** Patients’ demographic data, with lesion location, Digit span, Aachener Aphasie Test sub-tests as indexes of verbal impairment

Aphasics	Gender	Age	Education(years)	Occurrence of lesion (months)^a^	Digit span	**AAT—Aachener Aphasie Test**					Lesion location in left hemisphere and aetiology
						**TT**	**Rep**	**Wr. L**	**Den**	**Compr**	
01—ZA	F	53	13	53	6	3	145	89	112	114	Frontal/temporal stroke
02—ZA	M	56	13	115	5	12	139	73	86	101	Extensive temporal stroke
03—ZP	M	64	18	198	4.5	16	145	80	113	114	Frontal/temporal/parietal stroke (putamen, claustrum, external capsule)
04—ME	F	44	8	120	4	5	131	71	117	109	Extensive temporal/parietal AVM
05—TR	M	49	18	12	5	3	142	75	117	111	Deep-seated nuclear haemorrhage
06—BA	M	43	13	14	5	19	116	61	83	103	Parietal/occipital haemorrhagic stroke
07—BS	F	19	11	26	3	26	140	81	110	103	Capsular haematoma and extensive temporal/parietal AVM
08—PP	F	52	11	18	5	1	134	82	111	119	Frontal/temporal/parietal stroke
09—FB	M	73	8	22	4	2	139	86	112	112	MCA and ACA haemorrhagic stroke
10—BA	M	22	8	38	5	1	138	85	115	112	Cerebral peduncle and inner capsula head trauma
11—CR	F	42	8	7	5	6	139	83	113	110	Frontal/temporal/parietal vascular ischaemia
12—ZL	M	40	18	6	4	3	123	83	116	114	MCA haemorrhagic stroke
13—TE	F	55	8	12	8	0	149	89	120	120	Frontal/temporal ischemic stroke
14—CM	F	51	8	6	4	13	149	85	114	112	Frontal operculum/anterior insula ischaemia
15—TE	F	41	11	39	3	29	124	80	74	100	Carotid-ophthalmic aneurysm
16—RA	M	64	7	31	4	25	122	48	104	89	Deep frontal/parietal haemorrhage
17—VM	F	71	5	39	3	24	79	28	97	97	Frontal/parietal perisylvian stroke

^a^Notwithstanding all patients were ≥ 6 months post CVA, the the interval since the lesion was highly variable. We therefore correlated the time since onset with AAT subtests, behavioural (RTs and mean error rates) and electrophysiological (N150 and N350 components) data, and no significant correlations were found.

## References

[b1] Cappa S. Neuroimaging of recovery from aphasia. Neuropsychol Rehabil 10, 365–376 (2000).

[b2] Price C. J. & Crinion J. The latest on functional imaging studies of aphasic stroke. Curr Opin Neurol 18, 419–429 (2005). 10.1097/01.wco.0000168081.76859.c1 16003120

[b3] Belin P. *et al.* Recovery from non-fluent aphasia after melodic intonation therapy. Neurology 47, 1504–1511 (1996). 8960735 10.1212/wnl.47.6.1504

[b4] Musso M. *et al.* Training-induced brain plasticity in aphasia. Brain 122, 1781–1790 (1999). 10468516 10.1093/brain/122.9.1781

[b5] Thomas C., Altenmüller E., Marckmann G., Kahrs J. & Dichgans J. Language processing in aphasia: changes in lateralization patterns during recovery reflect cerebral plasticity in adults. Electroencephalogr Clin Neurophysiol 102, 86–97 (1997). 9060859 10.1016/s0921-884x(96)95653-2

[b6] Weiller C. *et al.* Recovery from Wernicke’s aphasia: a positron emission tomographic study. Arch Neurol 37, 723–732 (1995). 10.1002/ana.410370605 7778845

[b7] Karbe H. *et al.* Brain plasticity in post-stroke aphasia: what is the contribution of the right hemisphere? Brain Lang 64, 215–230 (1998). 9710490 10.1006/brln.1998.1961

[b8] Kessler J., Thiel A., Karbe H. & Heiss W. D. Piracetam improves activated blood flow and facilitates rehabilitation of poststroke aphasic patients. Stroke 31, 2112–2116 (2000). 10978039 10.1161/01.str.31.9.2112

[b9] Müller R.-A. *et al.* Language organization in patients with early and late left-hemisphere lesion: a PET study. Neuropsychologia 37, 545–557 (1999). 10340314 10.1016/s0028-3932(98)00109-2

[b10] Warburton E., Price C. J., Swinburn K. & Wise R. J. S. Mechanisms of recovery from aphasia: Evidence from positron emission tomography studies. J Neurol Neurosurg Psychiatry 66, 155–161 (1999). 10071093 10.1136/jnnp.66.2.155 PMC1736204

[b11] Cardebat D. *et al.* Behavioral and neurofunctional changes over time in healthy and aphasic subjects. A PET language activation study. Stroke 34, 2900–2907 (2003). 14615626 10.1161/01.STR.0000099965.99393.83

[b12] Jodzio K., Drumm D. A., Nyka W. M. & Gasecki D. The contribution of the left and right hemispheres to early recovery from aphasia: a SPECT prospective study. Neuropsychol Rehabil 155, 588–604 (2005). 16381142 10.1080/09602010443000137

[b13] Mimura M. *et al.* Prospective and retrospective studies of recovery in aphasia. Changes in cerebral blood flow and language function. Brain 121, 2083–2094 (1998). 9827768 10.1093/brain/121.11.2083

[b14] Ohyama M. *et al.* Role of nondominant hemisphere and undamaged area during word repetition in post-stroke aphasics. Stroke 27, 897–903 (1996). 8623110 10.1161/01.str.27.5.897

[b15] Rosen H. J. *et al.* Neural correlates of recovery from aphasia after damage to left inferior frontal cortex. Neurology 55, 1883–1894 (2000). 11134389 10.1212/wnl.55.12.1883

[b16] Saur D. *et al.* Dynamics of language reorganization after stroke. Brain 129, 1371–1384 (2006). 16638796 10.1093/brain/awl090

[b17] Thompson C. K. Neuroplasticity: Evidence from aphasia. J Commun Disord 33, 357–366 (2000). 11001162 10.1016/s0021-9924(00)00031-9 PMC3086401

[b18] Angrilli A., Elbert T., Cusumano S., Stegagno S. & Rockstroh B. Temporal dynamics of linguistic processes are reorganized in aphasics’ cortex: an EEG mapping study. Neuroimage 20, 657–666 (2003). 14568442 10.1016/S1053-8119(03)00395-1

[b19] Breier J. I. *et al.* Spatiotemporal patterns of language-specific brain activity in patients with chronic aphasia after stroke using magnetoencephalography. Neuroimage 23, 1308–1316 (2004). 15589095 10.1016/j.neuroimage.2004.07.069

[b20] Cornelissen K. *et al.* Adult brain plasticity elicited by anomia treatment. J Cogn Neurosci 15, 444–481 (2003). 12729495 10.1162/089892903321593153

[b21] Dobel C. *et al.* Slow event-related brain activity of aphasic patients and controls in word comprehension and rhyming tasks. Psychophysiology 39, 747–758 (2002). 12462503 10.1111/1469-8986.3960747

[b22] Friederici A. D., von Cramon D. Y. & Kotz S. A. Language related brain potentials in patients with cortical and subcortical left hemisphere lesions. Brain 122, 1033–1047 (1999). 10356057 10.1093/brain/122.6.1033

[b23] Hagoort P., Brown C. M. & Swaab T. Lexical–semantic event related potential effects in patients with left hemisphere lesions and aphasia, and patients with right hemisphere lesions without aphasia. Brain 119, 627–649 (1996). 8800953 10.1093/brain/119.2.627

[b24] Hagoort P., Wassenaar M. & Brown C. Real-time semantic compensation in patients with agrammatic comprehension: electrophysiological evidence for multiple-route plasticity. Proc Natl Acad Sci USA 100, 4340–4345 (2003). 12642675 10.1073/pnas.0230613100 PMC153094

[b25] Pulvermüller F., Mohr B. & Lutzenberger W. Neurophysiological correlates of word and pseudo-word processing in well-recovered aphasics and patients with right-hemispheric stroke. Psychophysiology 41, 584–591 (2004). 15189481 10.1111/j.1469-8986.2004.00188.x

[b26] Spironelli C., Angrilli A. & Pertile M. Language plasticity in aphasics after recovery: evidence from slow evoked potentials. Neuroimage 40, 912–922 (2008). 18252272 10.1016/j.neuroimage.2007.12.012

[b27] Swaab T., Brown C. & Hagoort P. Spoken sentence comprehension in aphasia: event-related potential evidence for a lexical integration deficit. J Cogn Neurosci 9, 39–66 (1997). 23968179 10.1162/jocn.1997.9.1.39

[b28] ter Keurs M., Brown C. M., Hagoort P. & Stegeman D. F. Electrophysiological manifestation of open- and closed-class words in patients with Broca’s aphasia with agrammatic comprehension. An event-related brain potential study. Brain 122, 839–854 (1999). 10355670 10.1093/brain/122.5.839

[b29] Bates E. *et al.* Voxel-based lesion-symptom mapping. Nat Neurosci 6, 448–450 (2003). 12704393 10.1038/nn1050

[b30] Clark D. G., Charuvastra A., Miller B. L., Shapira J. S. & Mendez M. F. Fluent versus nonfluent primary progressive aphasia: a comparison of clinical and functional neuroimaging features. Brain Lang 94, 54–60 (2005). 15896383 10.1016/j.bandl.2004.11.007

[b31] Damasio H., Grabowski T. J., Tranel D., Hichwa R. D. & Damasio R. A. A neural basis for lexical retrieval. Nature 380, 499–505 (1996). 8606767 10.1038/380499a0

[b32] Nestor P. J. *et al.* Progressive non-fluent aphasia is associated with hypometabolism centred on the left anterior insula. Brain 126, 2406–2418 (2003). 12902311 10.1093/brain/awg240

[b33] Brem S. *et al.* Neurophysiological signs of rapidly emerging visual expertise for symbol strings. Neuroreport 16, 45–48 (2005). 15618888 10.1097/00001756-200501190-00011

[b34] Cohen L. *et al.* The visual word form area: spatial and temporal characterization of an initial stage of reading in normal subjects and posterior split-brain patients. Brain 123, 291–307 (2000). 10648437 10.1093/brain/123.2.291

[b35] Dehaene S. Electrophysiological evidence for category-specific word processing in the normal human brain. Neuroreport 6, 2153–2157 (1995). 8595192 10.1097/00001756-199511000-00014

[b36] Liu Y. & Perfetti C. A. The time course of brain activity in reading English and Chinese: an ERP study of Chinese bilinguals. Hum Brain Mapp 18, 167–175 (2003). 12599274 10.1002/hbm.10090 PMC6871937

[b37] Puce A., Allison T., Asgari M., Gore J. C. & McCarthy G. Differential sensitivity of human visual cortex to faces, letterstrings, and texture – a functional magnetic resonance imaging study. J Neurosci 16, 5205–5215 (1996). 8756449 10.1523/JNEUROSCI.16-16-05205.1996 PMC6579313

[b38] Rossion B., Joyce C. A., Cottrell G. W. & Tarr M. J. Early lateralization and orientation tuning for face, word, and object processing in the visual cortex. Neuroimage 20, 1609–1624 (2003). 14642472 10.1016/j.neuroimage.2003.07.010

[b39] Salmelin R., Service E., Kiesilä P., Uutela K. & Salonen O. Impaired visual word processing in dyslexia revealed with magnetoencephalography. Ann Neurol 40, 157–162 (1996). 8773596 10.1002/ana.410400206

[b40] Schendan H. E., Ganis G. & Kutas M. Neurophysiological evidence for visual perceptual categorization of words and faces within 150 ms. Psychophysiology 35, 240–251 (1998). 9564744

[b41] Spironelli C. & Angrilli A. Influence of Phonological, Semantic and Orthographic tasks on the early linguistic components N150 and N350. Int J Psychophysiol 64, 190–198 (2007). 17363097 10.1016/j.ijpsycho.2007.02.002

[b42] Spironelli C. & Angrilli A. Developmental aspects of automatic word processing: language lateralization of early ERP components in children, young adults and middle-aged subjects. Biol Psychol 80, 35–45 (2009). 18343558 10.1016/j.biopsycho.2008.01.012

[b43] Tarkiainen A., Helenius P., Hansen P. C., Cornelissen P. L. & Salmelin R. Dynamics of letter string perception in the human occipitotemporal cortex. Brain 122, 2119–2131 (1999). 10545397 10.1093/brain/122.11.2119

[b44] Fiez J. A. & Petersen S. E. Neuroimaging studies of word reading. Proc Natl Acad Sci USA 95, 914–921 (1998). 9448259 10.1073/pnas.95.3.914 PMC33816

[b45] Dehaene S., Le Clec’H G., Poline J. B., Le Bihan D. & Cohen L. The visual word form area: a prelexical representation of visual words in the fusiform gyrus. Neuroreport 13, 321–325 (2002). 11930131 10.1097/00001756-200203040-00015

[b46] McCandliss B. D., Cohen L. & Dehaene S. The visual word form area: expertise for reading in the fusiform gyrus. Trends Cogn Sci 7, 293–299 (2003). 12860187 10.1016/s1364-6613(03)00134-7

[b47] Allison T., Puce A. & McCarthy G. Category-specific excitatory and inhibitory processes in human extrastriate cortex. J Cogn Neurosci 13, S103 (2001). 10.1152/jn.00202.2002 12424319

[b48] Allison T., Puce A. & McCarthy G. Category-sensitive excitatory and inhibitory processes in human extrastriate cortex. J Neurophysiol 88, 2864–2868 (2002). 12424319 10.1152/jn.00202.2002

[b49] Spironelli C., Penolazzi B., Vio C. & Angrilli A. Cortical reorganization in dyslexic children after phonological training: evidence from early evoked potentials. Brain 133, 3385–3395 (2010). 20688811 10.1093/brain/awq199

[b50] Meinzer M. *et al.* Intensive language training enhances brain plasticity in chronic aphasia. BMC Biol 2, 20 (2004). 15331014 10.1186/1741-7007-2-20 PMC515310

[b51] Szelies B., Mielke R., Kessler J. & Heiss W.-D. Prognostic relevance of quantitative topographical EEG in patients with poststroke aphasia. Brain Lang 82, 87–94 (2002). 12174818 10.1016/s0093-934x(02)00004-4

[b52] Spironelli C. & Angrilli A. EEG delta band as a marker of brain damage in aphasic patients after recovery of language. Neuropsychologia 47, 988–994 (2009). 19027029 10.1016/j.neuropsychologia.2008.10.019

[b53] Bentin S., Muochetant-Rostaing Y., Giard M. H., Echallier J. F. & Pernier J. ERP manifestations of processing printed words at different psycholinguistic levels: time course and scalp distribution. J Cogn Neurosci 11, 235–260 (1999). 10402254 10.1162/089892999563373

[b54] Angrilli A., Dobel C., Rockstroh B., Stegagno L. & Elbert T. EEG brain mapping of phonological and semantic tasks in Italian and German languages. Clin Neurophysiol 111, 706–716 (2000). 10727922 10.1016/s1388-2457(99)00308-9

[b55] Elbert T., Dobel C., Angrilli A., Stegagno L. & Rockstroh B. Word vs. task representation in neural networks. Behav Brain Sci. 22, 286–287 (1999).

[b56] Penolazzi B., Spironelli C., Vio C. & Angrilli A. Altered hemispheric asymmetry during word processing in dyslexic children: an event-related potential study. Neuroreport 17, 429–433 (2006). 16514371 10.1097/01.wnr.0000203350.99256.7d

[b57] Spironelli C. & Angrilli A. Language lateralization in Phonological, Semantic and Orthographic tasks: A slow evoked potential study. Behav Brain Res. 175, 296–304 (2006). 17045661 10.1016/j.bbr.2006.08.038

[b58] Spironelli C., Manfredi M. & Angrilli A. High-beta EEG band as marker of brain damage and functional recovery in aphasic patients. Cortex 49, 2650–2660 (2013). 23810123 10.1016/j.cortex.2013.05.003

[b59] Spironelli C., Penolazzi B. & Angrilli A. Dysfunctional hemispheric asymmetry of theta and beta EEG activity during linguistic tasks in developmental dyslexia. Biol Psychol 77, 123–131 (2008). 17997211 10.1016/j.biopsycho.2007.09.009

[b60] Spironelli C., Penolazzi B., Vio C. & Angrilli A. Inverted EEG theta lateralization in dyslexic children during phonological processing. Neuropsychologia 44, 2814–2821 (2006). 16876830 10.1016/j.neuropsychologia.2006.06.009

[b61] Spironelli C., Angrilli A., Calogero A. & Stegagno L. Delta EEG band as a marker of left hypofrontality in schizophrenia patients. Schizophr Bull 37, 757–767 (2011). 19933713 10.1093/schbul/sbp145 PMC3122275

[b62] Hagoort P. On Broca, brain, and binding: a new framework. Trends Cogn Sci. 9, 416–423 (2005). 16054419 10.1016/j.tics.2005.07.004

[b63] Bookheimer S. Functional MRI of language: new approaches to understanding the cortical organization of semantic processing. Annu Rev Neurosci. 25, 151–188 (2002). 12052907 10.1146/annurev.neuro.25.112701.142946

[b64] Caramazza A., Hillis A., Rapp B. & Romani C. The multiple semantics hypothesis: multiple confusions? Cogn Neuropsychol 7, 161–189 (1990).

[b65] Martin A., Wiggs C. L., Ungerleider L. G. & Haxby J. V. Neural correlates of category-specific knowledge. Nature 379, 649–652 (1996). 8628399 10.1038/379649a0

[b66] Abdullaev Y. G. & Posner M. I. Time course of activating brain areas in generating verbal associations. Psychol Sci. 8, 56–59 (1997).

[b67] Cobianchi A. & Giaquinto S. Event-related potentials to Italian spoken words. Electroencephalogr Clin Neurophysiol 104, 213–221 (1997). 9186236 10.1016/s0168-5597(96)96602-2

[b68] Thompson-Schill S. L., D’Esposito M., Aguirre G. K. & Farah M. J. Role of left inferior prefrontal cortex in retrieval of semantic knowledge: a reevaluation. Proc Natl Acad Sci USA 94, 14792–14797 (1997). 9405692 10.1073/pnas.94.26.14792 PMC25116

[b69] Kiehl K. *et al.* Neural pathways involved in the processing of concrete and abstract words. Hum Brain Mapp 7, 225–233 (1999). 10408766 10.1002/(SICI)1097-0193(1999)7:4<225::AID-HBM1>3.0.CO;2-P PMC6873335

[b70] Geschwind N. The organization of language and the brain. Science 170, 940–944 (1970). 5475022 10.1126/science.170.3961.940

[b71] Angrilli A. *et al.* Schizophrenia as failure of left hemispheric dominance for the phonological component of language. PLoS ONE 4, e4507 (2009). 19223971 10.1371/journal.pone.0004507 PMC2637431

[b72] Spironelli C., Angrilli A. & Stegagno L. Failure of language lateralization in schizophrenia patients: an ERP study on early linguistic components. J Psychiatry Neurosci 33, 235–243 (2008). 18592042 PMC2441888

[b73] Spironelli C., Bergamaschi S., Mondini S., Villani D. & Angrilli A. Functional plasticity in Alzheimer’s disease: effect of cognitive training on language-related ERP components. Neuropsychologia 51, 1638–1648 (2013). 23685197 10.1016/j.neuropsychologia.2013.05.007

[b74] Elbert T., Pantev C., Wienbruch C., Rockstroh B. & Taub E. Increased cortical representation of the fingers of the left hand in string players. Science 270, 305–307 (1995). 7569982 10.1126/science.270.5234.305

[b75] Taub E., Uswatte G. & Elbert T. New treatments in neurorehabilitation founded on basic research. Nat Rev Neurosci 3, 228–236 (2002). 11994754 10.1038/nrn754

[b76] Taub E., Uswatte G. & Pidikiti R. Constraint-induced movement therapy: a new family of techniques with broad application to physical rehabilitation – a clinical review. J Rehabil Res Dev 36, 237–251 (1999). 10659807

[b77] Kurland J., Pulvermüller F., Silva N., Burke K. & Andrianopoulos M. Constrained versus unconstrained intensive language therapy in two individuals with chronic, moderate-to-severe aphasia and apraxia of speech: behavioral and fMRI outcomes. Am J Speech Lang Pathol 21, S65–S87 (2012). 22294409 10.1044/1058-0360(2012/11-0113)

[b78] Luzzatti C. *et al.* L’Aachener Aphasie test (AAT-II): Proprietà psicometriche della versione italiana. Arch Psicol Neurol Psichiatria 48, 480–519 (1987).

[b79] Luzzatti C. *et al.* Nuovi dati normativi per la versione italiana dell’Aachener Aphasie test (AAT). Arch Psicol Neurol Psichiatria 55, 1086–1131 (1994).

[b80] Damasio H. & Damasio A. Lesion Analysis in Neuropsychology. New York: Oxford University Press (1989).

[b81] Oldfield R. C. The assessment and analysis of handedness: The Edinburgh Inventory. Neuropsychologia 9, 97–113 (1971). 5146491 10.1016/0028-3932(71)90067-4

[b82] Oostenveld R. & Praamstra P. The five percent electrode system for high-resolution EEG and ERP measurements. Clin Neurophysiol 112, 713–719 (2001). 11275545 10.1016/s1388-2457(00)00527-7

[b83] Grech R. *et al.* Review on solving the inverse problem in EEG source analysis. J Neuroeng Rehabil 5, 25 (2008). 18990257 10.1186/1743-0003-5-25 PMC2605581

[b84] Hassan M., Dufor O., Merlet I., Berrou C. & Wendling F. EEG source connectivity analysis: from dense array recordings to brain networks. PLoS ONE 9, e105041 (2014). 25115932 10.1371/journal.pone.0105041 PMC4130623

[b85] Bortolini V., Tagliavini C. & Zampolli A. Lessico di frequenza della lingua italiana contemporanea Milano: Aldo Garzanti Editore (1972).

[b86] Berg P. & Scherg M. Dipole models of eye movements and blinks. Electroencephalogr Clin Neurophysiol 79, 36–44 (1991). 1713550 10.1016/0013-4694(91)90154-v

[b87] Berg P. & Scherg M. A multiple source approach to the correction of eye artifacts. Electroencephalogr Clin Neurophysiol 90, 229–241 (1994). 7511504 10.1016/0013-4694(94)90094-9

[b88] Pascual-Marqui R. D. Standardized low resolution brain electromagnetic tomography (sLORETA): technical details. Methods Find Exp Clin Pharmacol 24D, 5–12 (2002). 12575463

[b89] Talairach J. & Tournoux P. Coplanar Stereotaxic Atlas of the Human Brain. Three-Dimensional Proportional System: An Approach to Cerebral Imaging. New York: Thieme Medical (1988).

